# MtRGF3 peptide activates defense responses and represses the expressions of nodulation signaling genes in *Medicago truncatula*: Activation of defense response by MtRGF3p

**DOI:** 10.3724/abbs.2023056

**Published:** 2023-06-28

**Authors:** Qiong Li, Dandan Shan, Wenjia Zheng, Yawen Wang, Zhiyin Lin, Huibo Jin, Anqi Ding, Junhui Yan, Liangliang Yu, Li Luo

**Affiliations:** Shanghai Key Laboratory of Bio-energy Crops School of Life Sciences Shanghai University Shanghai 200444 China

Root meristem growth factors (RGFs) were first discovered in *Arabidopsis thaliana* and regulate root development, including primary root growth, lateral root formation and root gravitropism, *etc*. [ 1‒ 3] . RGF belongs to a 13-amino-acid peptide family, which usually contains conserved aspartate (D1), tyrosine (Y2), proline (P9 and P10), histidine (H12) and asparagine (N13) residues. Among these residues, the second tyrosine residue (Y2) is modified by a sulfate group, and the tenth proline residue (P10) is hydroxylated [ 1‒ 3] . The active peptide is processed from a precursor that consists of a signal peptide, a variant region and a conserved C-terminal domain. The peptide is recognized by the leucine rich repeat (LRR) receptor kinases (RGFR) and then regulates the activities of the AP transcription factors TLPs in *A.*[ 4‒ 6] . Nine genes of *AtRGF* or *AtGLV* were identified in *A*. *thaliana*, and 15 corresponding genes were reported in the model legume *Medicago truncatula* [7]. Among these genes from *M*. *truncatula*, *MtRGF3* is abundantly expressed in leaves and is inducibly transcribed in roots by inoculation of the symbiont *Sinorhizbium meliloti* containing the *nodC* gene (required for nodulation factor biosynthesis). Reverse genetic analysis showed that overexpression of *MtRGF3* in the transgenic roots suppresses symbiotic nodulation of *M*. *truncatula*, while the RNAi increases the number of root nodules, suggesting that *MtRGF3* encodes a suppressor peptide of symbiotic nodulation in *M*. *truncatula* [7]. The MtRGF3 peptide (MtRGF3p) from chemical synthesis suppresses nodule primordium initiation, infection thread formation and root nodule development of *M*. *truncatula* seedlings on agar plates [7], consistent with the reverse genetic data. However, the molecular mechanism by which MtRGF3p suppresses symbiotic nodulation of *M*. *truncatula* is elusive.

Ethylene, an important hormone in higher plants, can increase plant defense responses under biotic and abiotic stresses. The precursor of ethylene biosynthesis, 1-aminocyclopropanecarboxylic acid (ACC), was used for the treatment of *M*. *truncatula* seedlings. The transcript level analysis from quantitative reverse transcription-PCR (qRT-PCR) showed that the expression of *MtRGF3* was repressed by 1 μM ACC ( Figure 1A), suggesting that *MtRGF3* may be associated with plant defense responses. The exopolysaccharide I (succinoglycan) from *S*. *meliloti* was reported to suppress host defense responses and to promote the symbiosis of *M*. *truncatula-S*. *meliloti* [8]. The inducible expression of MtRGF3 was observed in *M*. *truncatula* seedlings inoculated with the *exoY*210 mutant (the EPSI minus mutant) compared with seedlings inoculated with the wild-type Sm1021 ( Figure 1B), suggesting that MtRGF3 is associated with plant defense responses. To further test this hypothesis, NTB (nitrotetrazolium blue) staining was used to analyze superoxide anion levels in *M*. *truncatula* roots treated with the peptide, since reactive oxygen species (ROS) bursts act as a defense response in plants. The results showed that 10 μM of MtRGF3p increased the level of superoxide anion in the vascular bundles of *M*. *truncatula* roots compared with the random peptide ( Figure 1C). After inoculation of the symbiont Sm1021, the level of superoxide anion in the roots treated with the random peptide or MtRGF3p exhibited a decreasing trend ( Figure 1C). These observations indicated that MtRGF3p induces oxidative bursts at the early stage of *M*. *truncatula* symbiotic nodulation, which is suppressed by the inoculation of Sm1021.

**Figure 1:**
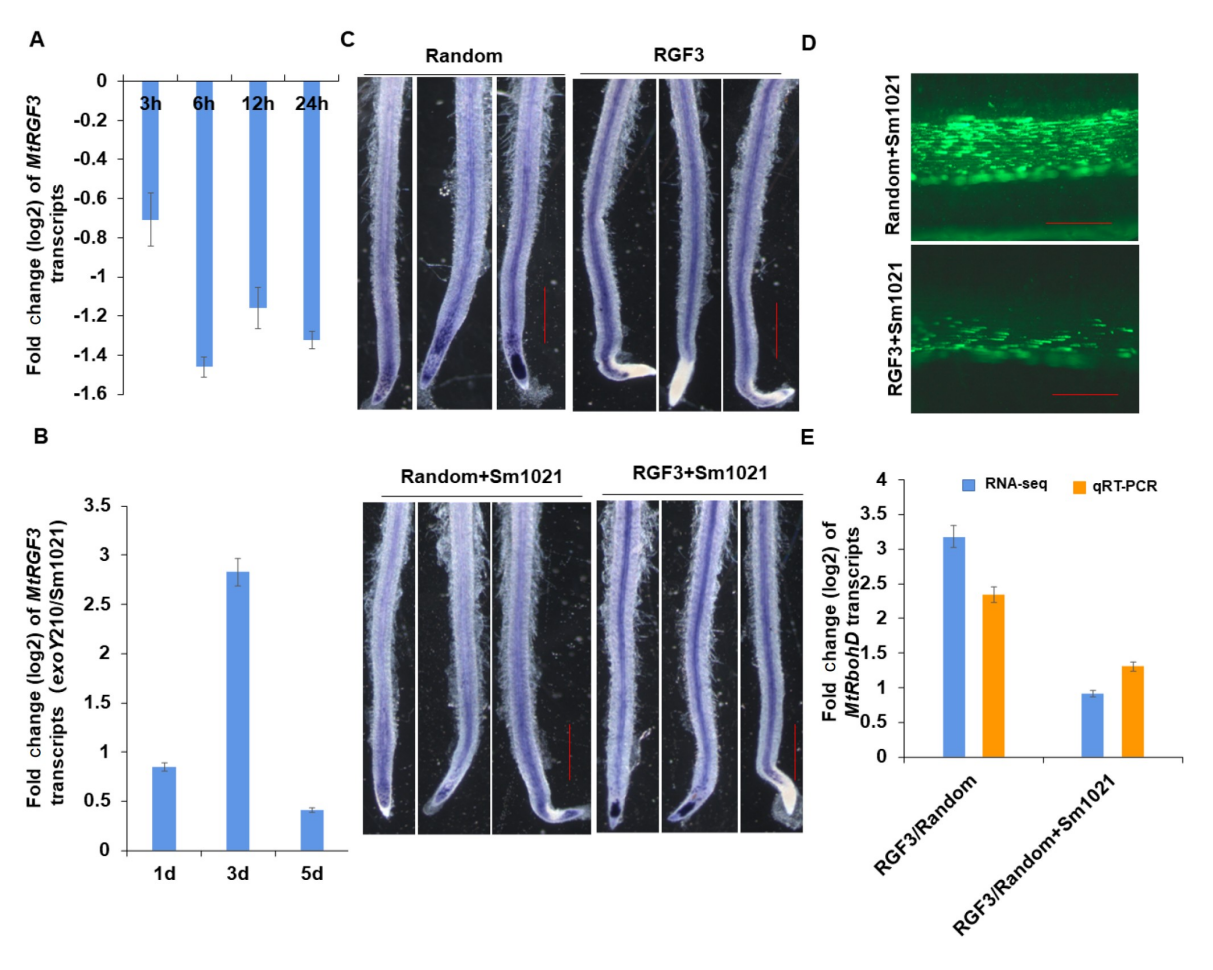
Figure 1 Defense responses of *M*. *truncatula* roots are activated after treatment with MtRGF3p (A) Expression of MtRGF3 was repressed by treatment with 1 μM ACC. (B) Expression of MtRGF3 was induced by the exoY mutant compared with Sm1021. (C) Levels of superoxide anion in M. truncatula roots stained by the NBT solution. Scale bar: 1 cm. (D) Colonies of Sm1021/pHC60 (the plasmid carrying a constitutively expressed gfp) on the roots. More than 40 M. truncatula seedlings were cultured on FM without combined nitrogen sources in the assays for each treatment. Scale bar: 100 μm. (E) Induction of MtRbohD in roots treated with MtRGF3p revealed by RNA-seq and quantitative RT-PCR. M. truncatula A17 seedlings were grown on FM medium with exogenous addition of 1 or 10 μM of the peptide. N>40 independent roots were used per peptide treatment in each experiment. Data are shown as the mean±SE.

The oxidative burst would affect rhizobium colonization on the host root surface, the first step of rhizobia infecting host legume plants. To verify this possibility, GFP-labelled Sm1021 was used to observe rhizobial colonies on inoculated root surfaces of *M*. *truncatula*. Fluorescence microscopy observations indicated that fewer colonies of Sm1021 (35 colonies/cm) were formed on the root surfaces of the *M*. *truncatula* seedlings treated with 10 μM MtRGF3p than on those treated with the random peptide (119 colonies/cm; Figure 1D). These results confirmed that MtRGF3p suppressed *S*. *meliloti* colonization on host roots by inducing the oxidative burst, consistent with data of infection thread and nodule formation [7].

The high-level accumulation of superoxide anion radicals in roots treated with MtRGF3p suggests that the expression of at least one *RbohD* (respiratory burst oxidase homologue D, one of the NADH oxidases generating superoxide anions) gene may be induced in *M*. *truncatula*. From the RNA-seq data, the induction of *MtRbohD* (Mtr3g098350) was observed in the roots treated with 1 μM MtRGF3p, but it was attenuated by inoculation with Sm1021 ( Figure 1E). This result was reconfirmed by qRT-PCR in the roots of *M*. *truncatula* treated with MtRGF3p ( Figure 1E).

Interestingly, among the differentially expressed genes of *M*. *truncatula* roots treated with 1 μM MtRGF3p compared with the random peptide from the RNA-seq transcriptome, all 5 *PR5* (pathogenesis-related protein 5) genes, 9 of 13 *WRKY* (transcription factors participate in response to biotic or abiotic stresses) genes, and 36 of 60 *R* (pathogen resistance protein) genes were inducibly expressed in the roots treated with MtRGF3p, while their induction was suppressed by the inoculation with Sm1021 ( Figure 2A and Supplementary Table S1). These results indicated that a broad defense response or innate immune genes are boosted by MtRGF3p in *M*. *truncatula*, so this peptide may be a defense or immunity signal in the plant to control rhizobium infection.

**Figure 2:**
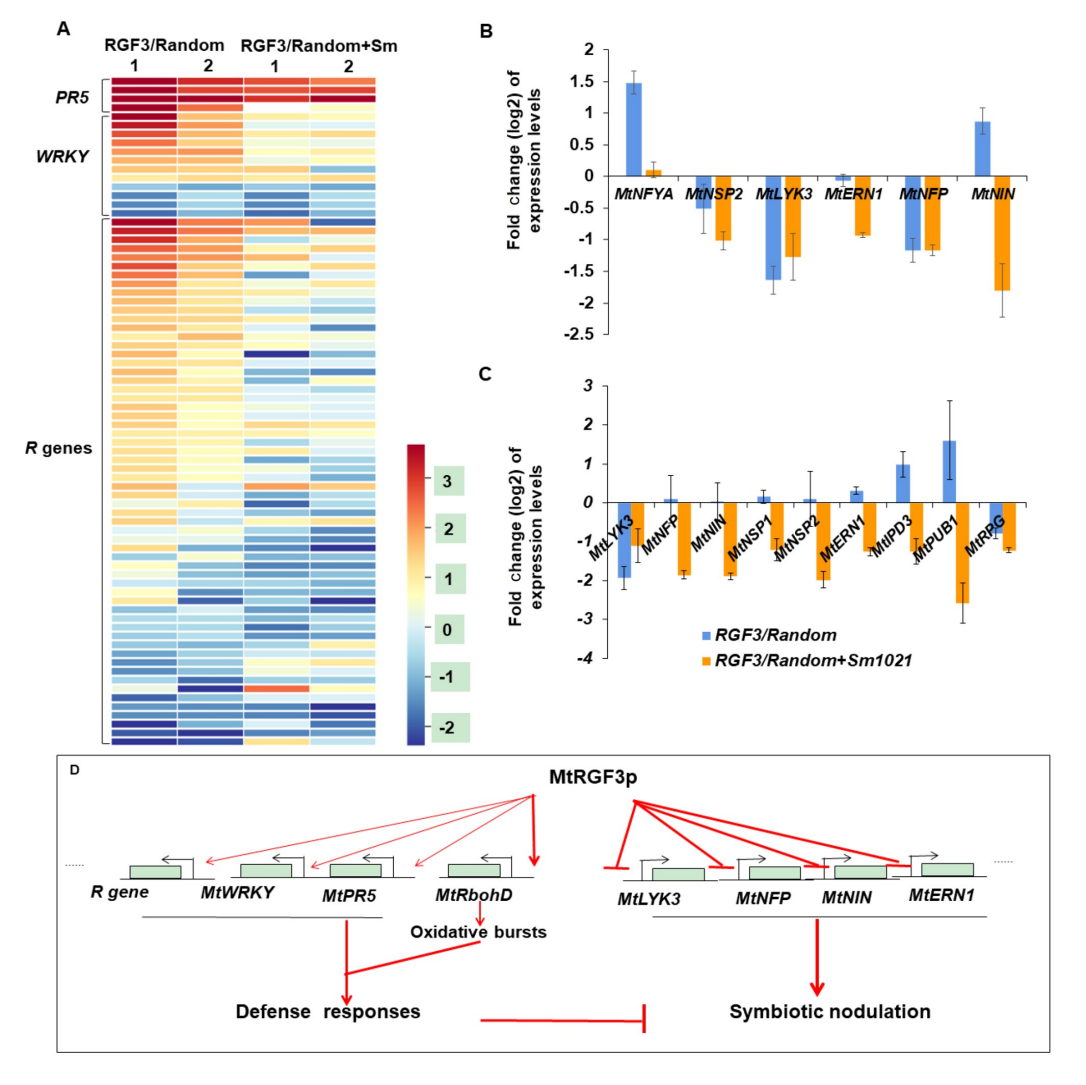
Figure 2 Gene differential expression in *M*. *truncatula* roots treated with MtRGF3p (A) Differential expression of pathogen resistance-associated genes in M. trucatula roots treated with 1 μM MtRGF3p from RNA-seq analysis. Two individual sequencing assays were performed in the study. Expressions of key nodulation signaling genes were repressed in M. truncatula roots treated with MtRGF3p as revealed by RNA-seq analysis (B) and quantitative RT-PCR assays (C). M. truncatula A17 seedlings were grown on FM medium with exogenous addition of 1 μM of the peptide. N>40 independent roots were used per peptide treatment in each experiment. Data are shown as the mean±SE. (D) A possible model of MtRGF3p suppressing nodulation of M. truncatula via activation of defense responses.

Nodulation factors (NFs), a group of lipo-chito-oligosaccharides produced by rhiozobia, are the most important signal during legume symbiotic nodulation. NF is sensed by the LysM receptor kinase MtNFP/MtLYK3 and activates calcium spiking and several transcription factors, such as MtNIN, MtNSP1, MtNSP2 and MtERN1, to reprogram the expressions of downstream genes to regulate rhizobium infection and nodule development. Since MtRGF3p suppresses symbiotic nodulation of *M*. *truncatula* [7], the downregulation of the key genes of the NF signaling pathway is possible. By mining RNA-seq data, we found that the transcription of key nodulation signaling genes such as *MtNFP*, *MtLYK3*, *MtERN1*, *MtNSP2* and *MtNIN* was repressed by treatment with 1 μM MtRGF3p compared with the random peptide after inoculation with Sm1021 ( Figure 2B), which was reconfirmed by qRT-PCR ( Figure 2C). These results first indicate that MtRGF3p suppresses *M*. *truncatula* nodulation by repressing the NF signaling pathway genes.

In the present study, we first provide the possible mechanism by which MtRGF3p suppresses *M*. *truncatula* symbiotic nodulation. This peptide activates the plant defense/immune response to restrict rhizobium colonization and infection, while it attenuates NF signaling by repressing key gene expression to reduce the initiation of nodule primordia and infection ( Figure 2D). In fact, a negative feedback loop may be formed. NF induces the expression of *MtRGF3* to produce the active peptide [7], but the peptide subsequently downregulates the key genes of NF signaling ( Figure 2B,C), especially the LysM receptor kinase *MtNFP*/ *MtLYK3*, so that NF signal transduction is attenuated. At the same time, the peptide activates the defense/immune response to prevent colony formation of *S*. *meliloti* on *M*. *truncatula* roots ( Figure 1C‒E and Figure 2A) so that less NF signaling is generated. Although the LRR receptor kinases (RGFRs) of AtRGF1 have been reported in *A*. *thaliana*[ 4‒ 6] , the homologous genes of MtRGF3p have not yet been identified in *M*. *truncatula*. If the LRR receptor kinase of MtRGF3p is identified from *M*. *truncatula*, the precise signaling mechanism will be resolved in the future. Moreover, whether MtRGF3p is associated with autoregulation of nodulation in *M*. *truncatula* may be elucidated. The supernodulation mutant ( *sunn-1*, [9]) of *M*. *truncatula* will be introduced for treatment with the peptide. The possibility that peptide signaling may be linked to ethylene signal transduction also needs to be studied in the *skl* mutant background [10].

## Supporting information

23032supplementary_data-ll

## Supplementary Data

Supplementary data is available at *Acta Biochimica et Biophysica Sinica* online.
