# Supplementary material for: MtRGF3 peptide activates defense responses and represses the expressions of nodulation signaling genes in Medicago truncatula : Activation of defense response by MtRGF3p
Source: Acta Biochim Biophys Sin (Shanghai). 2023 Jun 28;55(8):1319–22. doi: 10.3724/abbs.2023056 (PMC10448048; doi:10.3724/abbs.2023056)
Supplement: 23032supplementary_data-ll [file 23032supplementary_data-ll.pdf]

Materials and Methods

Preparation of plant materials

Medicago truncatula (A17) seedlings were inoculated with *Sinorhizobium meliloti* 1021 or *exoY*210 (about 10^7 cells/plate) on FM liquid medium (containing 1 or 10 μM of the MtRGF3 peptide or 1 μM of ACC). The plants were grown in a greenhouse at 23°C with 16 h of light per day.

RNA extraction, qRT-PCR and RNA-seq

Total RNA was extracted from plant tissues using an RNAprep pure Plant kit (DP437; Tiangen Biotech Co., Ltd., Beijing, China). Synthesis of first-strand cDNA and real-time PCR were performed using the PrimeScript™ RT Reagent kit with gDNA Eraser and SYBR Green qPCR Master Mix-SYBR Advantage (RR037Q; TaKaRa, Dalian, China,). For gene expression analysis, qRT-PCR was performed according to the kit instructions (TaKaRa). The housekeeping gene is *MtACTIN*, and the primers are shown in Supplementary Table S2. RNA-seq was performed and the data were analyzed by Bohao Biotech (Shanghai, China). The protocol of RNA sequencing and data analysis can be seen on the website www.shbio.com..

In situ superoxide detection

To detect O_2^-, alfalfa roots were harvested at 14 dpi and stained with 0.5 mg/mL nitroblue tetrazolium (NBT; Sigma-Aldrich, St Louis, USA) in 0.1 M potassium phosphate at pH 7.0. Nodules were vacuum-infiltrated three times (5 min each) in NBT staining solution. After infiltration, the nodules were stained for 45 min at room temperature followed by a wash with distilled water. The nodules were rapidly immersed in the coagulation reagent containing 1.25 M trichloroacetaldehyde hydrate (Sinopharm Chemical Reagent Co. Ltd, Shanghai, China) and 25% glycerol for 1 h and then analyzed by optical microscopy.

Observation of rhizobium colonies on the root surface

Roots of seven-day-old *M. truncatula* seedlings grown on FM agar medium were

transferred to sterile dishes containing 15 mL of 1 mM $CaCl_2$ and 0.4% sucrose. Next, the seedlings were inoculated with an OD_{600} of 0.01 of the appropriate derivative strain of *S. meliloti* 1021 carrying the GFP reporter plasmid pHC60 (3). Twenty roots were inoculated per strain. After 2 days of incubation in the dark at room temperature, the root segments were rinsed and resuspended in fresh calcium chloride/sucrose solution. Ten roots were used to count the number of bacteria (colony-forming units (CFU) mL-1), and another ten roots were sealed under coverslips. Fluorescence microscopy was performed under a Nikon microscope.

Supplementary Table S1. Differential expression of defense-associated genes in *M. truncatula* roots treated with MtRGF3p

**Gene**	**family**	**RGF3P1_FPKM**	**RGF3P2_FPKM**	**RGF3PplusSim10211_FPKM**	**RGF3PplusSim10212_FPKM**	**Rundom1_FPKM**	**Rundom2_FPKM**	**RundomplusSim10211_FPKM**	**RundomplusSim10212_FPKM**	**RGF3/random-1**	**RGF3/random-2**	**RGF3/random+Sm1021-1**	**RGF3/random+Sm1021-2**
MTR_2g435310	PR5	10.54428591	3.006194258	4.256763789	2.261330625	2.721327025	1.237178715	5.117809818	2.413835972	1.954079152	1.280884321	-0.2657695	-0.094155702
MTR_4g120940	PR5	289.4204578	229.6364017	198.9101597	132.7259415	16.97101786	25.88110483	29.23511211	26.55806404	**4.092021902**	**3.149380244**	2.766342937	2.321228486
MTR_4g120970	PR5	259.9433713	180.4469247	179.8687696	210.1341332	17.09684245	24.98920048	27.41272899	28.92099818	**3.926395557**	2.852197907	2.714026851	2.861121274
MTR_6g033450	PR5	204.8795652	159.9359479	142.8779023	153.1651014	6.7193429	11.84344171	17.04345845	10.35015018	**4.930312131**	**3.755333947**	3.067492783	3.887364011
MTR_1g013790	WRKY family transcription factor	2.109409829	1.673111589	0.281190341	1.138037148	0.136121274	0.305753025	0	0.774648423	3.953874956	2.452094994		0.554934058
MTR_7g073	WRK	15.44042	5.8561	6.2956	4.30353	1.2111	1.886	3.2328445	2.60770	3.6723	1.63392	0.96	0.722

430	Y transcription factor	287	26793	15409	6224	03406	899611	56	0573	18309	9121	1543271	74439
MTR_8g032510	WRKY transcription factor	3.552984577	1.586159985	1.365263889	1.011610382	0.347806716	0.39068442	1.235576878	0.989828541	3.352673729	2.021462663	0.143995056	0.031403201
MTR_8g005750	WRKY transcription factor	20.83806654	17.56680447	11.7628166	10.75341084	3.14766556	5.355273628	5.861658862	4.383135748	2.726867256	1.713819582	1.004852641	1.294759071
MTR_0002s1250	WRKY transcription factor	10.09624185	8.330428415	4.487171833	5.307230376	1.898985923	3.066091198	3.348101	4.513703455	2.410517258	1.441990082	0.422463382	0.233647536
MTR_1g015140	WRKY family transcription factor	1.316964239	0.741326543	0.772464413	0.934478201	0.311565581	0.174987734	0.488279655	0.483704657	2.0796084	2.082855368	0.661760805	0.950034582
MTR_8g092010	WRKY family transcription factor	6.205219025	2.15739462	1.605587399	1.977826017	1.813436026	0.678982579	1.299160626	1.206448918	1.774756283	1.667843627	0.305521385	0.71314869
MTR_7g117200	WRKY family transcription factor	1.340601873	0.603674078	0.880601686	0.553464647	0.456720252	0.227998203	0.318099106	1.023995285	1.553498184	1.404747399	1.469013288	-0.887646002
MTR_8g005750	WRKY transcription factor	20.83806654	17.56680447	11.7628166	10.75341084	3.14766556	5.355273628	5.861658862	4.383135748	1.004852641	1.294759071	1.004852641	1.294759071
MTR_3g104750	WRKY	0.593040024	1.001372076	0.521738044	0.382503809	0.631348584	0.2363804	0.879450471	0.871099536	-0.75327669	-1.187363462	-0.75327	-1.1873634

	family transcription factor						9			5		6695	62
MTR_8g099350	WRKY family transcription factor	80.41359882	6.803316023	12.46264696	15.34135212	35.51148132	9.376063104	36.71720025	21.3337896	-1.558845538	-0.475714618	-1.558845538	-0.475714618
MTR_4g013260	WRKY transcription factor	12.7167445	0.840155429	2.451152146	0.924283504	2.316386056	1.332626478	7.260579846	1.447058984	-1.566624733	-0.64671639	-1.566624733	-0.64671639
MTR_5g074200	WRKY family transcription factor	0.964155707	0.285659584	0.119054319	0.36659793	0.6722707	0.701209238	0.421427944	0.745385001	-1.823666023	-1.023787148	-1.823666023	-1.023787148
MTR_3g464870	LRR and NB-ARC domain disease resistance protein	2.583680003	2.116266645	2.09431825	0.566982995	0.211188509	0.428809525	0.499021781	2.130911448	**3.612824156**	2.30311256	2.069305998	-1.910093271
MTR_2g035150	disease-resistance response protein	201.2029873	170.3892871	156.9814705	149.9201082	20.92368168	32.69288232	48.6111294	44.58492432	3.265443093	2.381786151	1.69123572	1.749566026
MTR_3g056410	LRR and NB-ARC domain disease	0.288717356	0.142208078	0.063505108	0.130365374	0.034152177	0.038362466	0.085636227	0.10604231	**3.0796084**	1.890236049	-0.4313486	0.297920727

	resistance protein												
MTR_8g073850	disease resistance-responsive_dirigent domain protein	4.2488765	3.223586727	3.900635363	1.81131792	0.699255388	0.785459777	2.191826724	0.67839141	2.60319004	2.037057495	0.831575389	1.416849982
MTR_2g014840	LRR and NB-ARC domain disease resistance protein	0.185386596	0.178879075	0.201933639	0.136669904	0.03759767	0.042225587	0.062839894	0.136149553	2.301821778	2.082795262	1.684128618	0.005503332
MTR_8g099135	disease resistance response protein	17.62080914	9.773832037	10.41029596	10.35423454	2.616822081	3.554474254	7.0196598	4.25106044	2.75139243	1.459288127	0.568538065	1.284326227
MTR_4g081250	disease resistance protein (TIR-NBS-LRR class)	0.088562309	0.119651814	0.041557448	0	0.016770265	0.03765633	0.105074906	0.052031957	2.400787351	1.667877972	-1.338239195	0
MTR_6g046900	NBS-LRR resistance protein	0.182805453	0.308738877	0.21446227	0.141497235	0.043272497	0.145747556	0.162675553	0.067163475	2.078786816	1.082915471	0.398726399	1.07502509
MTR_2g035170	disease-resista	72.46065102	75.18619688	51.57867674	54.06749646	21.66334369	32.005823	26.01578514	43.60835511	1.74194183	1.232133378	0.987387	0.31015698

	nce response protein						97					513	2
MTR_8g469090	disease resistance protein (TIR-NBS-LRR class)	1.016912474	1.36285048	0.795200242	0.649701194	0.320781843	0.617660273	0.63194913	0.782444519	1.66453112	1.141741844	0.331509764	-0.268212103
MTR_5g092310	disease resistance protein (TIR-NBS-LRR class)_ putative	3.344507366	1.31779273	1.225994442	0.776643858	1.186868708	0.53325646	1.63682673	1.289611702	1.494633371	1.30522203	-0.416949171	-0.731611655
MTR_8g073770	disease resistance-responsive_ dirigent domain protein	7.538831638	5.40613269	5.390773144	4.370051158	2.882967821	2.085628131	2.818136593	3.640848324	1.386786219	1.374114975	0.935750659	0.26337552 9
MTR_3g015260	disease resistance protein (CC-NBS-LRR class) family protein	0.022915786	0.017193845	0.008959138	0.00787877	0.007230816	0.008118118	0.009061005	0.022434558	1.664111484	1.082675042	-0.016311101	-1.509680404
MTR_6g078490	NB-ARC	0.523248839	0.428405902	0.297581641	0.24546954	0.270076396	0.1348317	0.206926423	0.18632866	0.95412965	1.667817868	0.524167	0.39769441

	domain protein						71					647	4
MTR_7g018670	LRR and NB-ARC domain disease resistance protein	0.216638707	0.292664618	0.330384263	0.178884849	0.082004367	0.138170831	0.205625096	0.159115136	1.401518385	1.082795262	0.684128618	0.168960114
MTR_8g020300	disease resistance protein (TIR-NBS-LRR class)	0.550807145	0.63832932	0.038008465	0.267533324	0.16864133	0.378828261	0.36520476	0.523545675	1.707589129	0.752756955	-3.264312909	-0.968596799
MTR_2g450830	disease resistance protein (TIR-NBS-LRR class)	0.427290881	0.502914579	0.296194038	0.52119149	0.183795109	0.22709876	0.299561808	0.513541881	1.217120155	1.146993545	-0.016311101	0.021331591
MTR_6g052750	LRR and NB-ARC domain disease resistance protein – putative	0.217841422	0.245171497	0.085167116	0.074896951	0.068737383	0.154344462	0.172270956	0.213266786	1.664111484	0.667637543	-1.016311101	-1.509680404
MTR_3g014040	LRR and	0.144959836	0.114241658	0.051016292	0.134652304	0.054871725	0.0616363	0.103192709	0.085188194	1.401518385	0.890236049	-1.01631	0.66051351

	NB-ARC domain disease resistance protein						26					1101	5
MTR_6g471150	NBS-LRR type disease resistance protein	0.042932251	0.04831847	0.02517714	0.022141069	0.020320175	0.022813689	0.025463408	0.031539053	1.079148983	1.082675042	-0.016311101	-0.510414413
MTR_3g033380	LRR and NB-ARC domain disease resistance protein	0.018791433	0.021148979	0.011020026	0.029083031	0.008894134	0.009985544	0.011145325	0.027595223	1.079148983	1.082675042	-0.016311101	0.075759099
MTR_8g020360	disease resistance protein (TIR-NBS-LRR class)_putative	1.664369872	1.049280116	0.741898653	1.065019882	0.582748292	0.67225435	0.592403883	1.051280545	1.514031302	0.64232078	0.324641024	0.018732644
MTR_3g058720	disease resistance protein (TIR-NBS-LRR class)_putativ	0.115606894	0.078082141	0	0	0.04376074	0.049155576	0	0	1.401518385	0.667637543	0	0

MTR_1g036860	e NBS-LRR type disease resistance protein	0.173296778	0.11704651	0.203296749	0.107304387	0.065598123	0.073685077	0.082243305	0.050933401	1.401518385	0.667637543	1.305616994	1.07502509
MTR_4g013350	disease resistance response protein	15.8202293	12.36650826	8.446461515	7.919880421	7.808680405	6.424536985	4.552190731	5.249992695	1.018619838	0.944773818	0.891786072	0.593163232
MTR_7g091110	LRR and NB-ARC domain disease resistance protein	0.29806688	0.356537349	0.240392502	0.307649197	0.141032502	0.198023782	0.353659155	0.273648911	1.0796084	0.848379507	-0.556967707	0.168960114
MTR_1g064040	NB-ARC domain protein	0.412081352	0.309273027	0.214833312	0.330762665	0.173301616	0.194666286	0.108637999	0	1.249644066	0.667877972	0.983688899	0
MTR_3g058670	disease resistance protein (TIR-NBS-LRR class)_ putativ	1.826878496	1.028325422	0.597562653	0.670798637	0.814520271	0.634827284	1.354680556	1.032135859	1.165358177	0.695860849	-1.180790805	-0.621681222
MTR_5g037610	e disease resistance protein (TIR-	2.83201417	2.408852813	1.476426668	1.49392748	1.131539311	1.672303923	1.120039114	2.403621657	1.323541777	0.526509179	0.398560586	-0.686099709

	NBS-LRR class)												
MTR_6g015585	disease resistance protein (TIR-NBS-LRR class)	0.304226814	0.513592016	0.535231269	0.31394805	0.143992965	0.323325038	0	0.670249399	1.079148983	0.667637543	0	-1.094172171
MTR_6g078780	disease resistance protein (TIR-NBS-LRR class)	0.950830424	0.713612015	1.487109634	1.308430194	0.299955861	0.786047696	0.376004572	0.465562457	1.664437847	-0.13947695	1.983688899	1.49079033
MTR_8g016480	disease resistance protein (TIR-NBS-LRR class)	1.899935994	1.353197945	0.864167033	0.840394937	0.733833951	1.442485835	1.932175057	1.139159144	1.372425259	-0.092184261	-1.16084371	-0.438829934
MTR_3g058710	disease resistance protein (TIR-NBS-LRR class)_putative	0.728603956	0.644447383	0.183146983	0.429710605	0.590963624	0.331908888	0.679174434	0.611568406	0.302065497	0.957275308	-1.890780218	-0.509148496
MTR_3g056590	NB-ARC domain disease resistance	3.030225664	1.111567744	1.198162174	0.808229663	1.256550598	1.158068507	2.120773864	1.950549476	1.269956471	-0.059124725	-0.823767602	-1.271043387

MTR_4g022950	protein NBS-LRR resistance	0.419040665	0.330242512	0.344107514	0.692028005	0.15861969	0.400892327	0.19886861	0.307773775	1.401518385	-0.279688953	0.791043822	1.168960114
MTR_8g016660	protein disease resistance protein (TIR-NBS-LRR class)	0.297390256	0.446267219	0.465069867	0.306842319	0.281419263	0.316059306	0.70553667	0.873583486	0.079636298	0.497712541	-0.601273601	-1.50944811 3
MTR_6g038720	disease resistance protein (NBS-LRR class) family protein	2.42025127	1.147227358	0.747133901	0.591595141	1.988932492	1.015429643	1.66245757	1.777589565	0.283162526	0.176061051	-1.153878788	-1.587240144
MTR_8g016440	disease resistance protein (TIR-NBS-LRR class)_ putative	0.22208445	0.12501258	0.16282934	0.057306017	0.091952414	0.265564569	0.47759024	0.367008032	1.272149022	-1.086989402	-1.52412677	-2.679053106
MTR_5g092220	toll-interleukin-resistance (TIR) domain protein	0.860321228	0.774806651	0.605488096	0.674827808	1.400289059	0.548648916	1.265693831	1.263764961	-0.70277733	0.497953083	-1.063757961	-0.905136842
MTR_4g017780	disease resistance	0.060017535	0.02251575	0.023464409	0.020634874	0.037856513	0.042523474	0.07119361	0.058757176	0.664842518	-0.917324958	-1.601273601	-1.509680404

	family protein _LRR protein												
MTR_7g069200	NB-ARC domain disease resistance protein	2.009871176	1.257017542	1.178863808	0.806670129	1.584995553	2.37365995	2.914561688	1.476245511	0.342624241	-0.917108487	-1.305881882	-0.8718819 34
MTR_3g435720	LRR and NB-ARC domain disease resistance protein	0.349089172	0.392991438	0.299235921	0.263288982	0.406581461	0.585106085	0.461961336	0.552236758	-0.219948827	-0.574200342	-0.626488739	-1.0686397 69
MTR_0573s0030	disease resistance protein (TIR-NBS-LRR class)	0.077960921	0.029247263	0.060959084	0.053634718	0.04917444	0.082855017	0.123304396	0.114505052	0.664842518	-1.502287459	-1.016311101	-1.0941721 71
MTR_5g092340	NB-ARC domain protein	0.597497084	0.044844242	0.180226064	0.017616794	0.28539794	0.169356335	0.297065313	0.091948378	1.065956747	-1.917067296	-0.720972496	-2.3838727 41
MTR_5g092520	toll_interleukin-like receptor–protein	0.701014671	0.526129103	0.411171041	0.422032716	1.271480386	0.558859833	1.039683107	1.201389239	-0.858992665	-0.087069634	-1.338333302	-1.5092768 99
MTR_3g022920	disease resistance	0.031001737	0.034891171	0.018180623	0.031992363	0.044005238	0.049421865	0.036774679	0.045526058	-0.505326203	-0.502287459	-1.016311101	-0.5089649 59

	protein (CC-NBS-LRR class) family protein												
MTR_5g037460	NB-ARC domain disease resistance protein	0.443048781	0.460405313	0.239871837	0.439680274	0.645015279	0.670157849	0.828920216	0.725889724	-0.541867782	-0.541596466	-1.788969463	-0.7232955 84
MTR_3g058840	disease resistance protein (TIR-NBS-LRR class)	0.208844069	0.160078236	0.166796806	0.110065728	0.344803331	0.217259757	0.448121	0.274147926	-0.723347543	-0.440643798	-1.425796674	-1.3165892 43
MTR_4g091450	NBS-LRR type disease resistance protein	0.107527095	0.145274124	0.100913169	0.177576467	0.183170617	0.297180 51	0.15309085	0.094777241	-0.76848786	-1.032561746	-0.601273601	0.905827829
MTR_3g011300	NBS-LRR type disease resistance protein	0.222452784	0.286204123	0.316846096	0.262393086	0.526259752	0.455996611	0.358187916	0.326766332	-1.242275911	-0.671978647	-0.176934428	-0.3165296 36
MTR_6g084370	disease resistance protein (TIR-NBS-LRR	0.09190185	0.258631029	0.485053338	0.28451542	0.260855037	0.439519973	0.327045644	0.134957888	-1.505082483	-0.765033354	0.5686514	1.075997542

	class)												
MTR_0137s0010	LRR and NB-ARC domain disease resistance protein	0.047754141	0.026872654	0.112019535	0.049280073	0.135545784	0.050751968	0.08496991	0	-1.505082483	-0.917324958	0.398726399	0
MTR_6g075870	disease resistance protein (TIR-NBS-LRR class)	0.179571916	0.10106306	0.263302914	0.1389999	0.276132128	0.357854976	0.426100788	0.230784464	-0.62079702	-1.824119274	-0.69447123	-0.731462262
MTR_1g037010	NBS-LRR type disease resistance protein	0.240579963	0.040118972	0.355368756	0.2390988789	0.202360271	0.274653506	0.063427034	0.143975061	0.249590412	-2.775256154	2.48614706	0.731787897
MTR_5g005550	disease resistance protein (TIR-NBS-LRR class)	0.014112361	0.015882878	0.016552073	0	0.053408869	0.029996564	0	0	-1.920119983	-0.917324958	0	0
MTR_1g007300	disease resistance protein (TIR-NBS-LRR class)	0.067979677	0.01913188	0.039875933	0.035084753	0.176927175	0.054198995	0.120987984	0.224707891	-1.379980242	-1.502287459	-1.601273601	-2.679134672
MTR_8g106450	disease resista	0.167517296	0.094266889	0.098238651	0.086392211	0.4754826	0.2670501	0.298066916	0.369061378	-1.50508248	-1.502287459	-1.60127	-2.0948876

	nce-responsive_dirigent domain protein						1			3		3601	16
MTR_0428s0030	disease resistance protein (TIR-NBS-LRR class)	0.025087898	0.056470781	0.073562591	0.038827914	0.130557162	0.119982704	0.074399009	0.128954921	-2.379618074	-1.087249959	-0.016311101	-1.731700704
MTR_7g070390	disease resistance response protein	0.518910021	0.511147418	0.456520788	0.87024927	2.148249063	3.447077448	1.385209787	2.191420371	-2.049604964	-2.753562374	-1.601352023	-1.332365652
MTR_3g011420	NBS-LRR type disease resistance protein	0.029974782	0.033735376	0.123048638	0.10825641	0.255242075	0.143354193	0.053334737	0.132076325	-3.090044984	-2.087249959	1.206081321	-0.286919434

Supplementary Table S2. Sequences of primers used for qRT-PCR

Gene	Forward primer (5′⟶3′)	Reverse primer(5′⟶3′)
MtActin	TCAATGTGCCTGCCATGTATGT	ACTCACACCGTCACCAGAATCC
MtLYK3	TCAACTCAAGATGCCTCAGG	TTCTGCATAATAGACAGCTCC
MtNFP	GCATTTCCTCACAACAATGTC	AGACTGAGCTCTGTATGCTAC
MtNIN	GCAATGTGGGGATTTAGAGAT	GGAAGATTGAGAGGGGAAG
MtERN1	GGAGATGGGTAGCTGAGATC	GTTCGAGTGTTCGAACCTCG
MtNSP1	TTTCATTACAATGACTATGG	TATTGGCATCAATTTGGTAC
MtNSP2	CAAATCATTGTCAAGCAAAGC	TTAATCAGCATCCTATAAATCAG
MtIPD3	CGTTTGTCGAAGAGAATATCAAC	CTATCAACAGGATCTCTGATTGG
MtPUB1	TCATCCATGATAAGAAGGATC	AATCCAATTGTATGAGACTCC
MtRPG	GAAGAAATTACAGGAAAGATGCG	GTGGACCTCTTCTCTTACTG
MtRbohD	ACATGGCTCAGGAGCAAGAC	TGAAGAAGGCGTGGAAAGTC

References

1. Q. Li, M. Li, D. Zhang, L. Yu, J. Yan, L. Luo, The peptide-encoding MtRGF3 gene negatively regulates nodulation of *Medicago truncatula*, Biochem Biophys Res Commun 523 (2020) 66-71.
2. Yu L, Huang L, Zeng S, Tang G, Wang S, Li N, Yan J, Luo L.Reactive oxygen species accumulation patterns of alfalfa root nodules identified using an optimized method. Acta Biochim Biophys Sin (Shanghai). 2019 Apr 1;51(4):448-451.
3. Cheng HP, Walker GC. Succinoglycan is required for initiation and elongation of infection threads during nodulation of alfalfa by Rhizobium meliloti. J Bacteriol. 1998 Oct;180(19):5183-91.
